# Ethyl Pyruvate Prevents Renal Damage Induced by Methylglyoxal-Derived Advanced Glycation End Products

**DOI:** 10.1155/2019/4058280

**Published:** 2019-10-14

**Authors:** Eunsoo Jung, Wan Seok Kang, Kyuhyung Jo, Junghyun Kim

**Affiliations:** ^1^Laboratory of Toxicology, Research Institute for Veterinary Science and College of Veterinary Medicine, Seoul National University, Seoul 08826, Republic of Korea; ^2^College Department of Oral Pathology, School of Dentistry, Chonbuk National University, Jeonju 54896, Republic of Korea; ^3^Clinical Medicine Division, Korea Institute of Oriental Medicine, Daejeon 34054, Republic of Korea

## Abstract

The renal accumulation of advanced glycation end products (AGEs) is a causative factor of various renal diseases, including chronic kidney disease and diabetic nephropathy. AGE inhibitors, such as aminoguanidine and pyridoxamine, have the therapeutic activities for reversing the increase in renal AGE burden. This study evaluated the inhibitory effects of ethyl pyruvate (EP) on methylglyoxal- (MGO-) modified AGE cross-links with proteins in vitro. We also determined the potential activity of EP in reducing the renal AGE burden in exogenously MGO-injected rats. EP inhibited MGO-modified AGE-bovine serum albumin (BSA) cross-links to collagen (IC_50_ = 0.19 ± 0.03 mM) in a dose-dependent manner, and its activity was stronger than aminoguanidine (IC_50_ = 35.97 ± 0.85 mM). In addition, EP directly trapped MGO (IC_50_ = 4.41 ± 0.08 mM) *in vitro*. In exogenous MGO-injected rats, EP suppressed AGE burden and MGO-induced oxidative injury in renal tissues. These activities of EP on the MGO-mediated AGEs cross-links with protein *in vitro* and *in vivo* showed its pharmacological potential for inhibiting AGE-induced renal diseases.

## 1. Introduction

Advanced glycation end products (AGEs) are generated in the human body and affect the function and structure of proteins. AGEs are slowly formed during the normal aging process, and some disease conditions such as diabetes accelerate this process [1]. AGEs naturally formed low levels in the body by protein or lipid glycation with sugars, and most of them are catabolized depending on the tissue anti-oxidative systems, macromolecular turnover, receptor-mediated degradation, and renal elimination [2]. However, a chronic increase of intracellular oxidative stress accelerates AGE formation and leads to accumulating it in an intracellular space. AGE formation is an irreversible reaction, and it can be cross-linked with proteins resulting in disturbed biological reaction; thus AGEs, are implicated in the pathogenic processes of various age-related diseases [3]. Particularly, matrix proteins such as collagen are properly cross-linked with AGEs in conditions of diabetes and aging [4, 5].

Methylglyoxal (MGO) is known as the major precursor for AGEs and generated as a side-product derived from glycolysis. MGO easily forms AGEs due to its high reactivity to cross-link with proteins [6]. MGO-derived protein modifications have been shown in human tissues [7]. Previous researches have shown that AGEs play an important role in the pathogenic processes of chronic kidney disease (CKD) [8], age-related renal injury [9], and diabetic nephropathy [10]. Oxidative stress or proapoptotic cytokine induced by the interaction of AGEs and its receptor was involved in the apoptosis of renal glomerular cell and [11] and podocytes [12]. AGEs induced mesangial expansion and proteinuria in animal experiments [13].

Aminoguanidine (AG), a well-known antiglycation agent, ameliorated diabetes-induced mesangial expansion and proteinuria in several animal experiments [14–16]. Nevertheless, the clinical trial of AG was discontinued due to serious adverse effects such as gastrointestinal disturbance and abnormalities in liver function [17]. Therefore, the development of an antiglycation agent is needed for patients with MGO or AGE-related renal insufficiency.

Some natural and synthetic compounds have been proposed as AGE inhibitors [18]. Ethyl pyruvate (EP) is considered safe for human consumption as a food additive [19]. Moreover, EP is a simple aliphatic ester derived from pyruvic acid and is more stable and safer than pyruvic acid to inhibiting the production of reactive oxygen species (ROS) and inflammation [7, 20]. EP has beneficial effects in various animal models of ischemia/reperfusion injury and hemorrhagic or endotoxic shock [21, 22]. EP has also shown a renoprotective effect in streptozotocin-induced diabetic rats [23]. Recently, Kim et al. reported that ethyl pyruvate prevented MGO-induced retinal vascular injury [24]. Despite the various effects of EP, it remains unclear whether EP has inhibitory effects on the glycation processes and its cross-links with proteins. Therefore, the aim of this study is to evaluate the inhibitory effect of EP on MGO-derived AGE formation in vitro and furthermore EP applied in exogenous MGO-injected rats to confirm the preventive effect on AGE accumulation and oxidative renal injury in vivo.

## 2. Materials and Methods

### 2.1. In Vitro Assay of the Cross-Linking of Glycated Proteins

AGE-modified bovine serum albumin (BSA) (1 *μ*g, TransGenic Inc., Kobe, Japan) was incubated with the presence or absence of EP or AG in collagen-coated 96-well plates for 4 hours. Collagen-AGE-BSA cross-linked complex was detected using a horseradish peroxidase-linked mouse anti-AGE antibody (6D12, Wako, Osaka, Japan). The IC_50_ concentration (*μ*g/ml) was calculated.

### 2.2. Chelating Assay of MGO

MGO (0.05 mM, Sigma, MO, USA) was incubated with the presence or absence of EP or AG for 30 min. The level of remaining MGO was then measured by 2,4-dinitrophenylhydrazin (2,4-DNPH) as previously described [25].

### 2.3. Animals and Experimental Design

All procedures using animals were approved by the Institutional Animal Care and Use Committee (IACUC approval No. 2015-088). Sprague-Dawley (SD) rats (~200 g) were randomized into five groups of 6 rats: Group 1—normal rat (NOR), Group 2—rats treated MGO by intraperitoneal (i.p.) injection (MGO), Group 3—rats treated MGO by i.p. injection and administered orally with 50 mg/kg of AG (AG), and Groups 4~6—rats treated MGO by i.p. injection and administered orally with three different doses of 10, 25, and 50 mg/kg of EP (EP10, EP25, and EP50) once a day for 2 weeks. For the oral gavage, AG and EP were dissolved in distilled water immediately before administration. We chose the i.p. routes to achieve a pathologically relevant plasma concentration of 2–5 mM MGO [26, 27]. According to a previous report [28], we gave 17.25 mg/kg (240 mmol/kg) of MGO by a single i.p. injection. Body weight and blood glucose levels were measured at the beginning and the end of the experiment. Blood glucose levels were analyzed using an automated analyzer (Wako, Tokyo, Japan).

### 2.4. Histopathology

Renal tissues were fixed in 10% neutralized formaldehyde and embedded in paraffin prior to preparing 4 *μ*m sections. The sections were stained with periodic acid-Schiff (PAS) reagent (Sigma, St. Louis, MO, USA) and counterstained with hematoxylin. The sections were examined by two experienced renal pathologists in a double-blinded manner.

### 2.5. Immunohistochemical Staining

At necropsy, the renal tissues were fixed with 10% formaldehyde and embedded in paraffin, and 5 *μ*m thick sections were prepared. Immunohistochemistry for *α*-smooth muscle actin (*α*-SMA), AGEs, and 8-hydroxy-2′-deoxyguanosine (8-OHdG) was performed with a monoclonal mouse anti-*α*-SMA (Santa Cruz, CA, USA), anti-AGEs (6D12, Wako, Osaka, Japan), and anti-8-OHdG antibody (Santa Cruz, CA, USA) according to a previously reported method [29]. Briefly, deparaffinized sections were hydrated and treated with 1% H_2_O_2_ in methanol prior to incubation with primary antibodies for 1 h at room temperature. Signal detection for *α*-SMA and 8-OHdG was achieved using the Envision kit (DAKO, Carpinteria, CA, USA) and visualized by 3,3′-diaminobenzidine tetrahydrochloride (DAB) chromogen. For the detection of AGEs, the sections were incubated using an Envision kit (DAKO, CA, USA) and visualized using 3-amino-9-ethylcarbazol (AEC) chromogen. As a negative control, tissue sections were incubated with serum from nonimmunized animals, instead of the primary antibody. The immunohistochemical signal intensity was analyzed in twenty randomly selected glomeruli from each rat using ImageJ software (National Institutes of Health, Bethesda, MD, USA). The intensity of the positive stained area was calculated in 5 randomly selected areas at 100x magnification using ImageJ software (National Institutes of Health, Bethesda, MD, USA).

### 2.6. Statistical Analysis

All data were expressed as the mean ± standard error of the mean (SE). Differences between groups were determined by one-way ANOVA followed by Tukey's post -hoc test using Prism 6.0 (GraphPad, CA, USA).

## 3. Results

### 3.1. Inhibitory Activity of EP on AGE Cross-Linking with Rat Tail Tendon Collagen

**Figure 1:**
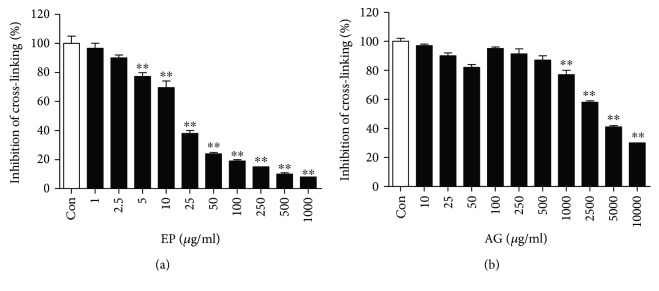
Inhibitory effect of EP (a) and AG (b) on the cross-links of AGE-BSA with collagen in vitro. All results are expressed the mean ± SE (*n* = 4). ^∗∗^*p* < 0.01 vs. the Con group.

The inhibition of AGE-BSA cross-linking to collagen at various concentrations of EP was tested. EP inhibited dose dependently the cross-linking of AGE-modified BSA with collagen (IC_50_ = 0.19 ± 0.03 mM, Figure 1(a)) and has 180 times stronger antiglycation activity AG (IC_50_ = 35.97 ± 0.85 mM, Figure 1(b)).

### 3.2. Methylglyoxal Scavenging Effect of EP

**Figure 2:**
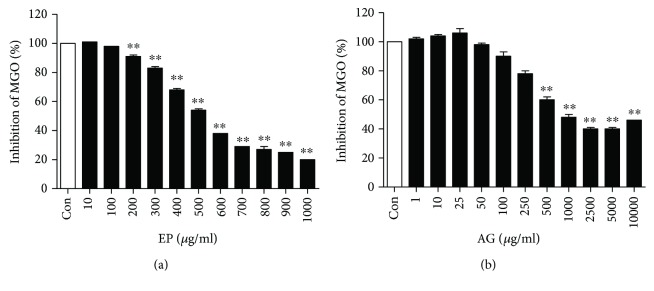
MGO chelating activity of EP (a) and AG (b). All results are expressed the mean ± SE (*n* = 4). ^∗∗^*p* < 0.01 vs. the Con group.

To investigate the role of EP as a potential AGE inhibitor, we tested whether EP can chelate MGO *in vitro*. As shown in Figure 2, EP chelated dose dependently MGO (IC_50_ = 4.41 ± 0.08 mM), and its chelating activity was 2 times stronger than AG (IC_50_ = 8.11 ± 0.78 mM).

### 3.3. Body Weight and Blood Glucose

Body weight and blood glucose levels are summarized in Table 1. No statistically significant differences in body weight or blood glucose levels were noted among all groups.

### 3.4. Effect of EP on Renal Histopathology in Exogenous MGO-Injected Rats

**Figure 3:**
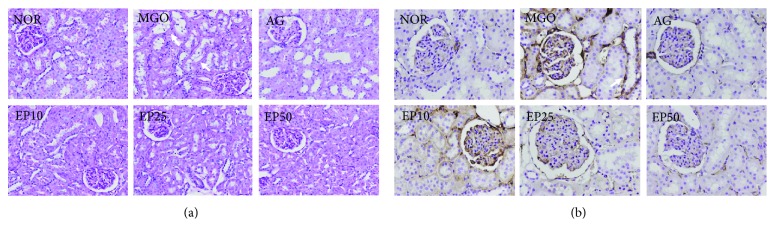
Renal histopathology. (a) Periodic acid-Schiff (PAS) staining of renal cortex. ×200 magnification. (b) Immunohistochemistry of *α*-SMA. ×200 magnification. NOR: normal control rats; MGO: exogenous MGO-injected rats; AG: MGO treated with aminoguanidine (50 mg/kg); EP10: MGO treated with EP (10 mg/kg); EP25: MGO treated with EP (25 mg/kg); EP50: MGO treated with EP (50 mg/kg).

A microscopic examination revealed that exogenous MGO-injected rats showed diffused mild degeneration of tubular epithelial cells. Affected tubules display both degenerative and regenerative changes including vacuole formation (Figure 3(a), arrow). At the same time, dilated tubules were filled with hyaline protein casts. Abnormalities were most prominent in tubular epithelial cells. Collecting ducts and glomerular tufts were uninjured or mildly degenerated. Vasculature was unaffected. However, the treatment of EP dose dependently inhibited these histopathological changes in exogenous MGO-injected rats. We next determined whether the renoprotection of EP is attributable to the regulation of *α*-SMA, an important fibrotic marker. Hence, the immunohistochemical staining of *α*-SMA was performed. As shown in Figure 3(b), *α*-SMA was almost undetectable in renal tissues in the control group, and MGO induced the marked expression of *α*-SMA in renal tubules (arrow). Furthermore, EP administration resulted in a dose-dependent decrease in the interstitial expression of *α*-SMA.

### 3.5. Effect of EP on Renal AGE Accumulations in Exogenous MGO-Injected Rats

**Figure 4:**
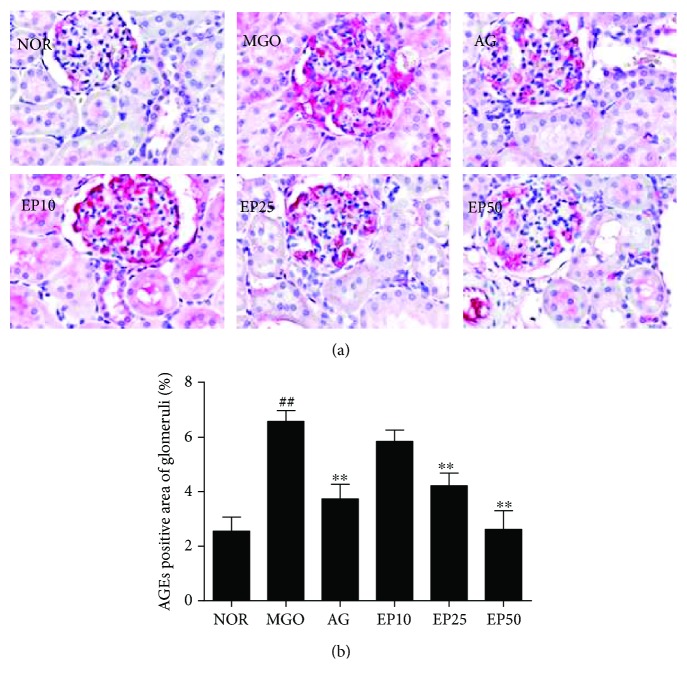
AGE accumulation in the renal glomeruli of exogenous MGO-injected rats. (a) Immunohistochemistry of AGEs. ×400 magnification. NOR: normal control rats; MGO: exogenous MGO-injected rats; AG: MGO treated with aminoguanidine (50 mg/kg); EP10: MGO treated with EP (10 mg/kg); EP25: MGO treated with EP (25 mg/kg); EP50: MGO treated with EP (50 mg/kg). (b) The immunohistochemically stained area was quantified at ×100 magnification. All data are expressed the mean ± SE (*n* = 6). ^##^*p* < 0.01 vs. the NOR group; ^∗∗^*p* < 0.01 vs. the MGO group.

**Figure 5:**
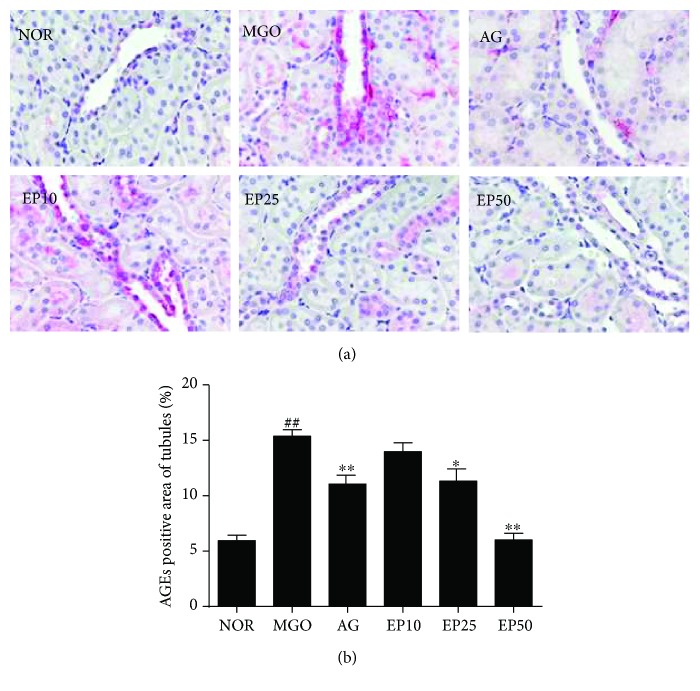
The effect of EP on AGE accumulation in the renal tubules of exogenous MGO-injected rats. (a) Immunohistochemistry of AGEs. ×400 magnification. NOR: normal control rats; MGO: exogenous MGO-injected rats; AG: MGO treated with aminoguanidine (50 mg/kg); EP10: MGO treated with EP (10 mg/kg); EP25: MGO treated with EP (25 mg/kg); EP50: MGO treated with EP (50 mg/kg). (b) The immunohistochemically stained area was quantified at ×100 magnification. All data are expressed the mean ± SE (*n* = 6). ^##^*p* < 0.01 vs. the NOR group; ^∗^*p* < 0.05 and ^∗∗^*p* < 0.01 vs. the MGO group.

To determine whether the intraperitoneal injection of exogenous MGO accelerates the renal accumulation of AGEs, we examine the immunohistochemical staining for AGEs. As shown in Figures 4 and 5, AGE accumulations were rarely found in the control group, but higher levels of those were found mainly in both glomeruli and tubules of MGO-injected rats. However, the treatment of EP dose dependently inhibited the renal accumulation of AGEs compared to the MGO group. The treatment with a high dose of EP (50 mg/kg) showed similar inhibition efficacy of AG (50 mg/kg) treatment.

### 3.6. Effect of EP on Renal Oxidative DNA Damage in Exogenous MGO-Injected Rats

**Figure 6:**
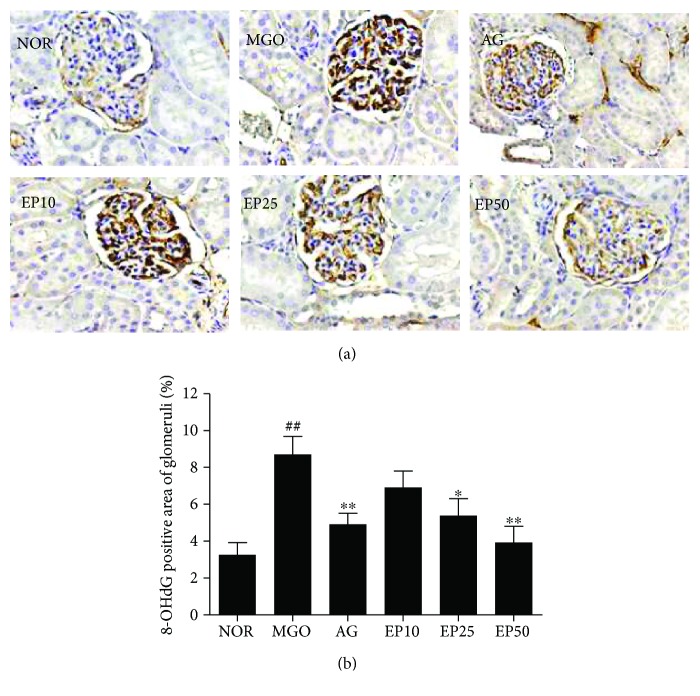
The effect of EP on oxidative DNA damage in the glomeruli of exogenous MGO-injected rats. (a) Immunohistochemical staining of 8-OHdG. ×400 magnification. NOR: normal control rats; MGO: exogenous MGO-injected rats; AG: MGO treated with aminoguanidine (50 mg/kg); EP10: MGO treated with EP (10 mg/kg); EP25: MGO treated with EP (25 mg/kg); EP50: MGO treated with EP (50 mg/kg). (b) The immunohistochemically stained area was quantified at ×100 magnification. All data are expressed the mean ± SE (*n* = 6). ^##^*p* < 0.01 vs. the NOR group; ^∗∗^*p* < 0.01 vs. the MGO group.

**Figure 7:**
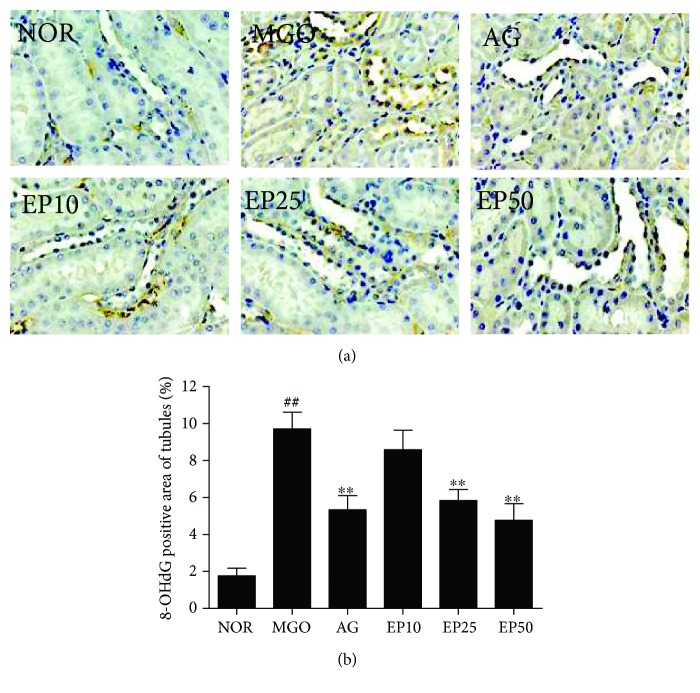
The effect of EP on oxidative DNA damage in the renal tubules of exogenous MGO-injected rats. (a) Immunohistochemical staining of 8-OHdG. ×400 magnification. NOR: normal control rats; MGO: exogenous MGO-injected rats; AG: MGO treated with aminoguanidine (50 mg/kg); EP10: MGO treated with EP (10 mg/kg); EP25: MGO treated with EP (25 mg/kg); EP50: MGO treated with EP (50 mg/kg). (b) The immunohistochemically stained area was quantified at ×100 magnification. All data are expressed the mean ± SE (*n* = 6). ^##^*p* < 0.01 vs. the NOR group; ^∗^*p* < 0.01 and ^∗∗^*p* < 0.01 vs. the MGO group.

To determine whether the intraperitoneal injection of exogenous MGO leads the oxidative renal injury, we examine the oxidative DNA damage in renal tissues using 8-OHdG immunostaining. 8-OHdG is known as a biomarker for measuring the oxidative damage of DNA [30]. As shown in Figures 6(a) and 7(a), the immunohistochemistry showed nuclear/perinuclear-positive staining of 8-OHdG in the renal mesangial cells, podocyte, and tubular epithelial cells. The immunoreactivity of 8-OHdG in the MGO group was significantly increased compared to the normal that in the control groups. However, EP treatment suppressed the expression of 8-OHdG compared to that observed in the MGO group (Figures 6(b) and 7(b)).

## 4. Discussion

Previous researches have revealed that AGEs play a crucial role in the pathogenic processes of various diseases including Alzheimer's disease, cardiovascular diseases, and diabetes [31–34]. AGE cross-links formed irreversible complexes when sugar permanently binds to the target protein, such as elastin and/or collagen. In this study, we showed that EP had the antiglycation activity *in vitro*, and we also found that MGO, one of the reactive carbonyls, was chelated by EP. EP showed a more potent antiglycation activity than AG. Moreover, EP had preventative activity against AGE formation and MGO-induced oxidative renal injury in the exogenous MGO-injected rats.

Many studies demonstrated that AGEs accumulated in many tissues in patients with diabetes. Their toxic activities under diabetic conditions have been demonstrated in several experimental studies [3]. MGO is a reactive carbonyl compound and a potent precursor of AGEs [35]. In particular, MGO-derived AGEs in the human plasma contribute to the development of various diseases such as diabetes [6], cancer [36], and cardiovascular diseases [33]. In the human body, there is no enzyme to destroy the AGE structure. Thus, AGEs are easily accumulated during the aging process [37]. The inhibition of MGO-derived AGEs and AGE cross-linking with protein can be an effective strategy for the prevention of the age-related disease. The chelating of MGO is known as a pharmacological strategy to inhibit MGO-derived AGE formation [38]. AG, as a well-known AGE inhibitor, prevents AGE accumulation by interacting with these reactive carbonyls. Other AGE inhibitors, including 2-isopropylidenehydrazono-4-oxo-thiazolidin-5-ylacetanilide (OPB-9195) and pyridoxamine, also inhibit AGE formation through the interaction with the reactive carbonyls as a MGO scavenger [39]. This study revealed that EP exhibited the MGO trapping ability. Collectively, these results suggest that the MGO chelating activity of EP could contribute to the inhibition of AGE accumulation in various tissues and maybe inhibit the development of AGE-related diseases.

To confirm the antiglycation activity of EP *in vivo*, EP was administered orally in exogenous MGO-injected rats. In the MGO-injected rats, AGEs largely accumulate in renal tissues and stimulate oxidative renal injury. AGEs are believed to play an important role in the major pathogenic processes of CKD, age-related renal failure, and diabetic nephropathy [8–10]. The inhibition of AGE burden by AGE inhibitors could ameliorate these renal diseases. In this study, EP prevented the renal AGE burden in the intraperitoneally exogenous MGO-injected rats in vivo. In addition, EP ameliorated the oxidative DNA damage induced by exogenous MGO. MGO has been shown to induce ROS generation [40]. AGEs also act as a source of ROS generation [5]. 8-OHdG is one of the major forms of DNA damage induced by ROS. In exogenous MGO-injected rats, AGE formation and 8-OHdG levels were highly increased in renal tissues. EP reversed these changes, indicating that EP is able to prevent MGO-induced oxidative stress by its ability to act as a MGO chelator.

**Figure 8:**
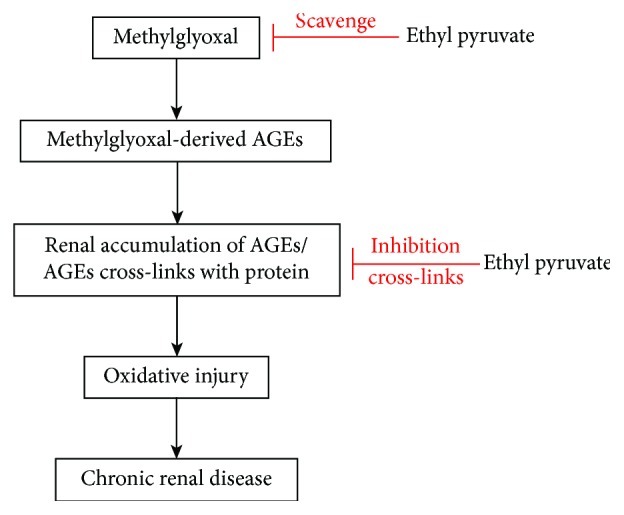
The mechanism of EP. EP can chelate MGO and AGE accumulation and improve the exogenous MGO-induced oxidative injury in renal tissues.

Many researches have demonstrated the effects of EP against cisplatin nephrotoxicity [41], endotoxemic shock [21] and ischemia-reperfusion injury through its antioxidant action in various experimental models [22]. Recently, a clinical trial was performed to identify the safety and pharmacological activity of EP against cardiopulmonary disease [19].  Figure 8 illustrates a mechanism by which MGO leads to renal injury and serves as a target for EP. Taken together, our results suggest that EP could be a prospect for preventative treatment in the pathogenic processes of chronic renal disease associated with AGE accumulation.

## Figures and Tables

**Table 1:** Physiological data of experimental rats.

		NOR	MGO	AG	EP-10	EP-25	EP-100
Body weight (g)	Initial	203.9 ± 22.7	207.9 ± 25.4	205.1 ± 20.3	205.5 ± 27.7	202.0 ± 31.5	203.9 ± 33.4
	Final	295 ± 39.7	305 ± 32.9	319 ± 42.1	299 ± 34.2	312 ± 41.5	308 ± 38.4

Blood glucose (mg/dl)	Initial	84.1 ± 11.0	89.5 ± 19.7	90.4 ± 14.8	91.2 ± 11.1	93.4 ± 17.7	95.1 ± 12.2
	Final	89.9 ± 12.1	95.2 ± 17.7	85.8 ± 18.7	87.5 ± 15.0	84.9 ± 18.8	97.5 ± 20.7

NOR: normal control rats; MGO: exogenous MGO-injected rats; AG: MGO treated with aminoguanidine (50 mg/kg); EP10: MGO treated with EP (10 mg/kg); EP25: MGO treated with EP (25 mg/kg); EP50: MGO treated with EP (50 mg/kg). All data are expressed as the mean ± SE (*n* = 6).

## Data Availability

The data used to support the findings of this study are available from the corresponding author upon reasonable request.
